# Going local: Evaluating guideline adherence and appropriateness of antibiotic prescribing in patients with febrile neutropenia at an academic teaching hospital

**DOI:** 10.1017/ash.2022.353

**Published:** 2023-01-09

**Authors:** Rachel Liu, Melissa R. Gitman, Andrew M. Morris, Miranda So

**Affiliations:** 1 Department of Pharmacy, Michael Garron Hospital, Toronto, Ontario, Canada; 2 Department of Pathology, Molecular and Cell-Based Medicine, Icahn School of Medicine at Mount Sinai, New York, New York, United States; 3 Sinai Health–University Health Network Antimicrobial Stewardship Program, Sina Health and University Health Network, Toronto, Ontario, Canada; 4 Department of Medicine, University of Toronto, Toronto, Ontario, Canada; 5 Leslie Dan Faculty of Pharmacy, University of Toronto, Toronto, Ontario, Canada

## Abstract

**Background:**

Febrile neutropenia (FN) is a medical emergency with significant morbidity and mortality for oncology patients, requiring comprehensive workup and timely antibiotic administration. We evaluated concordance with locally developed FN guidelines and outcomes of cancer patients admitted to general internal medicine at an academic teaching hospital.

**Methods:**

We conducted a retrospective observational cohort study of patients admitted between July 1, 2016, and June 30, 2017, for FN. Patients were classified as having low-risk or high-risk FN according to their malignancy and chemotherapy. Primary outcome was the proportion of patients receiving guideline-concordant antibiotics within 48 hours of admission to general internal medicine. Secondary outcomes were the proportion of patients in whom empirical antibiotics were active against pathogens isolated, rate of antibiotic-associated adverse events, and in-hospital mortality. We used logistic regression to model relationship between FN risk and guideline-concordant antibiotics.

**Results:**

Among 100 patients included, 34 (34%) were low-risk FN and 66 (66%) were high-risk. Proportion of guideline-concordant empirical antibiotics was significantly lower among low-risk FN patients than high-risk patients: 12 (35%) of 34 versus 47 (71%) of 66 (*P* = .001). Empirical antibiotics were active against 17 (94%) of 18 isolated pathogens. The mortality rate was 3%, and 16% of patients experienced antibiotic-associated adverse events. Hematological malignancy and infectious diseases–trained physician involvement were associated with guideline-concordant prescribing, with adjusted odds ratios of 3.76 (95% CI, 1.46–9.70; *P* = .006) and 3.71 (95% CI, 1.49–9.23; *P* = .005), respectively.

**Conclusions:**

Guideline concordance was low compared to published reports. Factors influencing appropriate antimicrobial prescribing in patients with FN warrant further exploration.

Febrile neutropenia (FN) is a medical emergency associated with significant potential morbidity and mortality for oncology patients.^1,2^ Signs and symptoms of infection are often absent or ambiguous due to diminished inflammatory responses in this patient population, but given the risk of infectious complications, timely administration of empirical antibiotics is recommended.^3^ The guideline from the Infectious Diseases Society of America (IDSA) for antimicrobial stewardship programs (ASP) has highlighted the value of ASP interventions in oncology patients to ensure appropriate selection of antibiotics tailored to local epidemiology while reducing antibiotic-associated adverse events associated with inappropriate use.^4–6^ The IDSA has emphasized the role of facility-specific, locally developed FN guidelines in this patient population.^6,7^ The implementation of institutional FN guidelines can help to optimize patient care by promoting best practices, improving patient outcomes, and reducing costs.^6,8^

The University Health Network (UHN) is a tertiary academic teaching hospital system affiliated with the University of Toronto. It includes 3 acute-care sites: Toronto General Hospital (TG), Princess Margaret Cancer Centre (PM), and Toronto Western Hospital. The UHN ASP created 2 FN guidelines based on local susceptibility pattern, patient population, and resources.^9,10^ One guideline is specific for patients with hematological malignancies such as acute leukemia and recipients of stem-cell transplant pre-engraftment, who are at high risk for infectious complications.^9^ The other guideline is specific for patients undergoing treatment for solid tumor, who are considered to be at low risk of infectious complications.^10^ The guideline for patients with hematological malignancies or stem-cell transplant recommends piperacillin-tazobactam plus tobramycin as empiric antimicrobial therapy. The guideline for the solid-tumor patients recommends cefazolin plus tobramycin. Empirical tobramycin is limited to a maximum of 72 hours for both guidelines while microbiological investigations are pending and diagnostic workup are underway. The inclusion of tobramycin is to ensure adequate gram-negative coverage, reflecting the epidemiology from the PM-specific antibiograms and Public Health Ontario’s provincial report. The prevalence of multidrug-resistant gram-negative rods in blood isolates is estimated to be between 20% and 25% in the local patient population.^11,12^ Meropenem is the empirical alternative for patients with self-reported allergy to penicillin and those with history of colonization or infection due to extended-spectrum β-lactamase–producing gram-negative bacilli.

In this study, we evaluated concordance with the FN guidelines and appropriateness of antibiotic prescribing among cancer patients admitted to TG general internal medicine (GIM) service. Although PM specializes in treating complex cancer patients, those who experience infectious complications are commonly admitted to TG. We aimed to identify opportunities to optimize febrile neutropenia management in GIM through this quality improvement initiative.

## Methods

### Design, setting, and study population

We conducted a retrospective observational cohort study of patients admitted to GIM between July 2016 and June 2017. The GIM service consisted of between 60 and 65 beds. Patients were identified by the UHN Department of Clinical Decision Support. We included patients with a primary discharge diagnosis of first-episode FN based on the *International Classification of Disease, Tenth Revision* (ICD-10) codes, who had a current oncology diagnosis and were currently receiving chemotherapy within 6 months prior to admission. We included patients admitted from TG emergency department or from PM’s rapid assessment clinic (TG and PM are located on the same street, across from one another). Patients were excluded if they were hematopoietic stem-cell transplant recipients in the postengraftment phase or if they were not undergoing chemotherapy. The study was approved by the UHN Institutional Review Board.

### Intervention

No direct antimicrobial stewardship interventions were underway in GIM during the study period. Following the launch of the FN guidelines (version 2016), presentations to introduce the guidelines were given by the ASP team to clinical teams across the UHN, including the TG GIM program. The guidelines are available on the institution’s intranet and on the ASP website.

### Data collection

Following eligible patients identified by the clinical decision support team, baseline patient demographics, clinical parameters including neutrophil counts and documented fever, imaging studies and microbiology data were collected through manual review of the institution’s electronic health record (EHR) system. An antimicrobial stewardship report generated by the ASP was provided to record the antibiotic regimens used.

### Definitions

FN was defined as per the IDSA guideline, with a composite of neutropenia as an absolute neutrophil count (ANC) of ≤0.5 × 10^9^/L or an ANC that is anticipated to decrease to <0.5 × 10^9^/L during the following 48 hours. Fever was defined as any reported or measured temperature of ≥38.3°C once or sustained temperature of ≥38°C for at least 1 hour.^2^ Oncology diagnoses were classified as follows: active solid tumor included breast, lung, colorectal, other gastrointestinal, gynecologic, genitourinary, head and neck, and low-risk lymphoma; active hematological malignancy included acute lymphoblastic leukemia (ALL), acute myeloid leukemia (AML), mixed ALL and AML, high-grade lymphoma (e.g., diffuse large B-cell lymphoma), chronic lymphoblastic leukemia (CLL), multiple myeloma, and myelodysplastic syndrome.^2,9,10^ We stratified patients as having low-risk or high-risk FN based on the IDSA guidelines, which were adopted in the UHN guidelines in 2014 and first updated in 2016. Low-risk patients were expected to have a short neutropenic period of ≤7 days; high-risk patients were anticipated to have prolonged neutropenia of >7 days; and profound neutropenia was defined as an absolute neutrophil count ≤0.1 × 10^9^ cells/L. In practice, patients undergoing treatment for solid tumor were managed under the low-risk guideline and patients with hematological malignancies were managed under the high-risk guideline. Age was categorized into <65 years or ≥65 years.

Guideline concordance was defined as the prescription of an empirical antimicrobial regimen that was consistent with the UHN FN guidelines recommendation according to the risk stratification of the patient. Empirical antimicrobials were considered appropriate if the regimen was active against the pathogen isolated from a microbiological culture and/or appropriate for the source of infection (e.g., pneumonia) according to the indication in the medical records. Antibiotic-associated adverse events were defined according to Tamma et al.^4^ Adverse events reported during hospitalization were categorized by body systems: gastrointestinal, dermatologic, musculoskeletal, hematologic, hepatobiliary, renal, cardiac, and/or neurologic.

Appropriateness of antimicrobial and guideline concordance were adjudicated by a study investigator (R.L.). For quality assurance, another clinician independently audited 20 randomly selected charts for accuracy of data collection.

### Outcomes

The primary outcome was the proportion of patients who received guideline-concordant antimicrobials within 48 hours of admission to GIM. We excluded antimicrobials administered while patients were in the emergency department because they had not been officially under the care of the attending internists. Secondary outcomes evaluated whether empirical antibiotics were active against the isolated pathogen or appropriate for the source of infection, factors associated with guideline concordance, incidence of nosocomial *Clostridioides difficile* infection, antibiotic-associated adverse events, length of stay under the GIM service, and in-hospital all-cause mortality.

### Statistical analysis

Baseline characteristics were reported with descriptive statistics. Outcome measures were compared using the χ^2^ test for categorical variables and the Wilcoxon rank-sum test for continuous variables. Univariable and multivariable logistic regression models were used to assess the association between FN risk and receipt of guideline-concordant antibiotics, adjusting for age, sex, type of cancer (e.g., hematological malignancy vs. solid tumor), and infectious diseases (ID) physician involvement. ID involvement was defined as having received ID specialist consultation or being under the care of an attending internist who was also a qualified ID consultant. Odds ratios (OR) and adjusted odds ratios (aOR) with 95% confidence intervals (CIs) for the primary outcome were reported. Model goodness-of-fit was assessed with the Akaike information criterion and likelihood ratio test. We defined *P* < .05 as statistically significant. Statistical analysis was completed using STATA version 16.0 software (StataCorp, College Station, TX).

## Results

### Patient demographics and clinical characteristics

In total, 100 patients met eligibility criteria to be included in the study. Baseline characteristics are presented in Table 1. Among them, 34 (34%) had low-risk FN and 66 (66%) patients had high-risk FN. Among the low-risk patients, duration of neutropenia was <7 days in 30 (88.2%) of 34 patients, although 15 (44%) had an absolute neutrophil count (ANC) of ≤0.1 × 10^9^ cells/L. In the high-risk group, 73% had ANC ≤0.1 ×10^9^ cells/L and 11% had neutropenia lasting 7 days or longer. Length of stay (LOS) was longer in the high-risk group (median, 6 days; interquartile range [IQR], 5) than the low-risk group (median 4, IQR 5; *P* = .0424). Duration of fever was shorter in the low-risk group, with a mean of 1.1 days (standard deviation, ±0.9 days), compared to 2.2 days (±1.9) in the high-risk group (*P* = .002).

**Table 1.** Patient Demographics

Risk of Febrile Neutropenia	Low Risk	High Risk
Unique admissions, no. (%)	34 (34)	66 (66)
**Sex, no. (%)**		
Male	21 (62)	47 (71)
Female	13 (38)	19 (29)
Age, median y (IQR)	61 (22)	63.5 (14)
**Primary cancer diagnosis, no. (%)**		
Prostate/Testicular	9 (26)	
Breast	6 (18)	
Ovarian/Endometrial	5 (15)	
Lung	3 (8)	
Low risk lymphoma	2 (6)	
Cholangiocarcinoma	1 (3)	
AML		25 (38)
High-risk lymphoma		20 (30)
ALL		6 (9)
MDS		5 (8)
MM		5 (8)
CLL		3 (4)
Other	8 (24)	2 (3)
**Absolute neutrophil count, no. (%)**		
>0.5 × 10^9^ cells/L	3 (9)	5 (8)
>0.1–0.5 × 10^9^ cells/L	16 (47)	13 (20)
Duration <7 d	15 (44)	12 (18)
Duration ≥7 d	1 (3)	1 (1)
≤0.1 × 10^9^ cells/L	15 (44)	48 (73)
Duration <7 d	15 (44)	41 (62)
Duration ≥7 d	0 (0)	7 (11)
Length of stay, median d (IQR)	4 (5)	6 (5)
Duration of fever, mean d (SD)	1.1 (0.9)	2.2 (1.9)
Self-reported allergies to penicillin, no. (%)	4 (12)	12 (18)

Note. IQR, interquartile range; SD, standard deviation; AML, acute myeloid leukemia; ALL, acute lymphoblastic leukemia; MDS, myelodysplastic syndrome; MM, multiple myeloma; CLL, chronic lymphoblastic leukemia.

### Primary outcome

All patients received antimicrobials within 48 hours of admission to GIM. Overall, the antimicrobial management of 59 (59%) of 100 patients was guideline concordant. In the low-risk group, 12 (35%) of 34 patients received guideline-concordant antimicrobials, and in the high-risk group 47 (71%) of 66 received guideline-concordant antimicrobials (*P* = .001) (Table 2). Patients in the low-risk group were less likely to have ID involvement compared to the high-risk group: 14 (41%) of 34 versus 43 (65%) of 66, respectively (*P* = .022). Factors associated with receiving guideline-concordant antimicrobials included type of cancer (hematological malignancy or pre-engraftment following stem-cell transplant versus solid tumor (adjusted odds ratio [aOR], 3.76; 95% confidence interval [CI], 1.46–9.70; *P* = .006) and involvement by an ID physician (aOR, 3.71; 95% CI, 1.49–9.23; *P* = .005) (Table 3).

**Table 2.** Guideline-Concordant Antimicrobial Prescribing

Parameter	Total		Low- Risk FN		High-Risk FN		*P* Value
	n/N	%	n/N	%	n/N	%	
Guideline concordant antimicrobial within 48 h	59/100	59	12/34	35	47/66	71	.001
ID involvement	57/100	57	14/34	41	43/66	65	.022
Source of infection identified	50/100	50	18/34	53	32/66	48	.67
Empirical antimicrobial active against pathogen or source	41/50	82	15/18	83	26/32	81	.85
**Targeted antimicrobial therapy**							
Microbiologically defined infections with bacterial isolates	17/18	94	7/7	100	10/11	91	n/a
Clinically defined infections	21/28	75	6/9	67	15/19	79	.48

Note. FN, febrile neutropenia.

**Table 3.** Factors Associated With Guideline-Concordant Prescribing

Variable	Univariate Analysis (OR, 95% CI)	*P* Value	Multivariable Analysis (OR, 95% CI)	*P* Value
Age (≥65 y vs ≤64 y)	1.74 (0.76–3.69)	.186	1.75 (0.68–4.52)	.241
Sex (women vs men)	0.48 (0.20–1.13)	.093	0.56 (0.21–1.50)	.250
Cancer type (malignant hematology or HSCT vs solid tumor)	4.53 (1.88–10.96)	.001	3.76 (1.46–9.70)	.006
ID involvement vs none	4.28 (1.83–10.01)	.001	3.71 (1.49–9.23)	.005

Note. OR, odds ratio; CI, confidence interval; HSCT, hematopoietic stem cell transplantation; ID, infectious diseases.

Regarding guideline-discordant antimicrobials received within the first 48 hours of admission to GIM, 9 (26%) of 34 patients in the low-risk group received piperacillin-tazobactam and tobramycin empirically, and 6 (18%) of 34 received piperacillin-tazobactam monotherapy, instead of cefazolin and tobramycin as recommended by the local guideline. In the high-risk group, 8 (12%) of 66 received piperacillin-tazobactam monotherapy, rather than piperacillin-tazobactam plus tobramycin as recommended by the local guideline.

### Secondary outcomes

Source of infection was identified in 50% of patients. The cause of fever was not identified in the other half of the study population. Of the 22 microbiologically defined infections, empiric antibiotic regimens were active against 17 (94%) of 18 bacterial pathogens isolated. Of the pathogens isolated, 8 of 22 were from blood specimens: 3 were gram-negative bacilli, 3 were gram-positive cocci, and 2 were polymicrobial. Of the 14 nonblood isolates, 7 were from urine specimens, all of which were gram-negative bacilli. Several multidrug-resistant organisms were identified. One vancomycin-resistant *Enterococcus* (VRE) and 1 extended-spectrum β-lactamase–producing (ESBL) *Escherischia coli* were isolated from a blood and urine source, respectively. Clinically defined infections occurred in 28 (28%) of 100 patients. Pneumonia was diagnosed in 21 patients, and 7 had skin and soft-tissue infections.

Hospital-onset of *C. difficile* infection occurred in 4 (4%) of 100. In-hospital all-cause mortality occurred in 3 (3%) of 100. Antibiotic-associated adverse drug events occurring in 16 (16%) of 100 patients with the most common being non–*C. difficile* associated diarrhea (n = 9), followed by dermatologic reaction (n = 3), and 1 patient each with hepatobiliary, gastrointestinal, renal, and neurological adverse events.

## Discussion

At our institution, the overall guideline concordance among FN patients in GIM at the Toronto General Hospital was low at 59%. Concordance was higher in the high-risk group (71%) than in the low-risk group (35%). Overall, guideline-concordant empirical antimicrobials were active against the clinical syndromes and isolated pathogens in FN patients, except for 1 VRE infection. A point-prevalence audit conducted at Princess Margaret Cancer Center in 2017 found FN guideline concordance to be 73% in patients with acute leukemia. The practice model and staffing at the malignant hematology units differed from that in the GIM units at Toronto General Hospital, which are clinical teaching units and patient care is provided by learners at various stages of training under the direct supervision of the attending internists. Furthermore, the ASP team conducted twice-weekly audit-and-feedback in the malignant hematology units at Princess Margaret. However, in TG GIM units, no direct antimicrobial stewardship interventions were conducted.

Our guideline-concordant antimicrobial prescribing was lower than the 79% reported in a database study of admission records between 2000 and 2010.^1^ However, our results shared common patterns with contemporaneous reports. Guideline concordance was 85.4% in a series of point-prevalence surveys conducted between 2014 and 2018 in a combination of academic and community hospitals in Australia.^13^ In this cohort, hematology patients were more likely to receive antimicrobials concordant with local guidelines than solid-tumor patients.^13^ Baugh et al^14^ conducted a case-vignettes survey among 107 oncologists of an academic cancer center and 44 physicians from the emergency department (ED) of an affiliated teaching hospital in 2015. These researchers reported that 96% of oncologists and 100% of ED physicians selected guideline-concordant antimicrobials for high-risk patients but that only 61% of oncologists and 23% of ED physicians selected appropriate plans for low-risk patients.^14^ In a retrospective cohort study by the same authors, guideline concordance was 70% among patients who presented with FN to the ED in 2010–2014, including 44 low-risk and 129 high-risk visits.^15^ There was a significant difference in the prevalence of guideline-discordant antimicrobials between low-risk and high-risk patients (98% vs 7%; risk ratio, 14; 95% CI, 7.5–26).^15^ Schuttevaer et al^16^ reported guideline adherence to be 395 (43.5%) of 909 in a retrospective cohort of FN patients who presented to the ED at a tertiary-care university hospital and had bloodstream infections between 2012 and 2017.^16^ In this study, over-treatment (spectrum too broad) with empirical antibiotics was not associated with survival benefit.^16^

Several factors may have contributed to poor guideline compliance in GIM. First, there were barriers to effective communication. UHN had a hybrid system of paper chart plus EHR that was not integrated with venues of communication, mainly paging and e-mail. GIM patients were located in several units, which were physical barriers of communication between prescribers and the ASP team. In addition, the GIM program is located at Toronto General Hospital—a different building from the Princess Margaret Cancer Centre—and did not receive scheduled audit-and-feedback interventions from the ASP team. No GIM pharmacist was dedicated to reviewing antimicrobial management in oncology patients or to liaising with the ASP team.

Second, there appeared to be a lack of familiarity of the FN guidelines among the GIM teams. Although the guidelines were on our institution’s intranet and the ASP website, active implementation strategies, education, and promotion efforts were not sustained in GIM units. In the survey conducted by Baugh et al^14^ in 2015, only 54% of oncologists and 26% of ED physicians were familiar with the IDSA guideline.^14^ Unfamiliarity with the guideline was associated with unnecessary empirical vancomycin in high-risk patients.^14^ In the Australian point-prevalence surveys, among hematology patients, 20.2% of meropenem and 11.3% of vancomycin were adjudicated as spectrum being too broad.^13^ In our study, the most common guideline-discordant prescribing in low-risk patients was the spectrum being too broad. Piperacillin-tazobactam monotherapy or in combination with tobramycin were prescribed instead of cefazolin and tobramycin. Because our recommended empirical therapy differed from the international guidelines, prescribers were not familiar with the rationale based on local epidemiology.^5^ Concerns regarding ototoxicity and nephrotoxicity from tobramycin exposure, albeit for ≤72 hours, may have contributed to a reluctance to prescribe.

Lastly, cancer type and ID physician involvement (as the primary team physician or formal consultation service) were independently associated with guideline-concordant antimicrobials. ID physicians may be more familiar with the guidelines, whereas patients with hematological malignancies were more likely to receive ID consultation service.^17^ Conversely, as demonstrated by Douglas et al,^13^ solid-tumor patients may have less exposure to oversight by ID specialists and ASP interventions. ID specialist involvement may promote guideline-driven practice by raising the awareness of other clinicians toward them.^14^

Since the conclusion of this study, TG GIM has installed a clinical pharmacist for oncology patients, who liaises with the ASP team and ID consultants. Recently, UHN replaced the hybrid system of paper chart and EHR system with an enterprise-wide integrated EHR system. The new system has built-in communication tools, documentation, and clinical decision support. Clinicians can access information on patients’ current cancer therapy directly. We anticipate that the new system will facilitate guideline-based prescribing and will improve the efficiency of evaluating prescribing practice.^14^ We are upgrading our guidelines to reflect a more nuanced approach to risk stratification, accounting for cancer diagnosis and up-to-date treatment.

The strengths of our study were intertwined with the quality improvement process. First, we focused on evaluating concordance and outcomes of patients admitted to GIM for FN, whereas many recent publications have concentrated on patients presented to the ED.^14–16,18^ Second, beyond process measures, we assessed appropriateness of guideline-recommended antimicrobials in the initial 48 hours after admission and clinical outcomes based on microbiological investigations. In comparison, others have focused on evaluating a single agent, most commonly vancomycin,^19,20^ route of antimicrobial administration,^15^ or admission according to disease burden score.^18^ There is increasing emphasis on auditing adherence to guidelines by standards-setting organizations such as the Center for Disease Control and the Joint Commission.^21–23^ In an era with growing prevalence of multidrug-resistant organisms, receiving inappropriate empirical antimicrobial treatment is associated with morbidity and mortality.^5,16^ Third, in our study population, patients were admitted over a 1-year period after guideline implementation. These results reflect the validity of the guideline recommendations. Previous publications have reported on data for cohorts of patients accumulated over long periods, often upwards of 5 years. The validity of those guidelines may wane over time owing to changes in the epidemiology of infectious complications, antimicrobial resistance, and advances in cancer treatment.^13,15,16,18–20^

Our study had several limitations. First, given its retrospective nature, the accuracy and validity of the data were limited by the quality of the documentation. A detailed examination of the prescriber’s clinical reasoning was precluded because the rationale and thought process behind antimicrobial prescribing was infrequently recorded. Second, there was overlap in duration and nadir of neutropenia between the high-risk and low-risk groups, suggesting that risk differentiation can be improved by incorporating details of cancer diagnosis and treatment. Third, as a single-center study, local factors contributing to guideline discordance may not apply elsewhere.

Further studies with quantitative and qualitative methods are needed to better understand why prescribers are not compliant with FN guideline recommendations and how those barriers can be removed.^24,25^ Serial point-prevalence audits on guidelines concordance and appropriateness of antimicrobial prescribing using an *a priori* adjudication framework with prescriber-specific data may be useful.^26^ In conclusion, we demonstrated that after developing local FN guidelines and making them available to prescribers, maintaining antimicrobial stewardship efforts, giving feedback, and evaluating adherence are imperative to ensuring that prescribers use antimicrobials according to best practices.

## Supplementary material

For supplementary material accompanying this paper visit https://doi.org/10.1017/ash.2022.353. click here to view supplementary material
