# Supplementary material for: Going local: Evaluating guideline adherence and appropriateness of antibiotic prescribing in patients with febrile neutropenia at an academic teaching hospital
Source: Antimicrob Steward Healthc Epidemiol. 2023 Jan 9;3(1):e3. doi: 10.1017/ash.2022.353 (PMC9879896; doi:10.1017/ash.2022.353)
Supplement: Supplementary file 1 [file S2732494X22003539sup001.docx]

**Supplementary Materials**

**Figure 1: Antimicrobials administered within 48 hours of admission on GIM**
